# The Capsule Increases Susceptibility to Last-Resort Polymyxins, but Not to Other Antibiotics, in Klebsiella pneumoniae

**DOI:** 10.1128/aac.00127-23

**Published:** 2023-03-13

**Authors:** Francesca D’Angelo, Eduardo P. C. Rocha, Olaya Rendueles

**Affiliations:** a Institut Pasteur, Université Paris Cité, CNRS UMR3525, Microbial Evolutionary Genomics, Paris, France

**Keywords:** multidrug resistance, antimicrobial compounds, capsule, *Klebsiella*, ESKAPE pathogen, colistin

## Abstract

The extracellular capsule is a virulence factor present in many facultative pathogens, but its role in antimicrobial resistance remains controversial. To shed light on this debate, we tested six antibiotics on four Klebsiella pneumoniae species complex strains. Noncapsulated strains exhibited increased tolerance to polymyxins, but not to other antibiotics, as measured using the MIC. Our results urge caution on the use of therapeutic agents that target the capsule and may result in selection for its inactivation.

## INTRODUCTION

In the last 20 years, the rise in multidrug resistant (MDR) infections has become a global health crisis that threatens the ability to treat many bacterial, viral, and fungal infections (1). The antimicrobial-resistant ESKAPE bacterial pathogens (Enterococcus faecium, Staphylococcus aureus, Klebsiella pneumoniae, Acinetobacter baumannii, Pseudomonas aeruginosa, and Enterobacter species) are recognized by the World Health Organization as capable of panresistance and are currently a key focus of public health research (2). Among the ESKAPE pathogens, Klebsiella pneumoniae causes a wide range of infections, mostly in immunocompromised individuals (3). Virulence factors in hypervirulent Klebsiella strains include adhesins, lipopolysaccharide, and iron-scavenging systems (4, 5), but the best known is the polysaccharide capsule, a feature common to all ESKAPE pathogens. The capsule protects the cell from the bactericidal action of host serum, impairs phagocytosis (6), and increases survival in the presence of biotic stresses, such as killing by the type VI secretion system (7). Some studies have asserted that the capsule protects against antimicrobial peptides (AMPs) (8, 9), small naturally occurring peptides with broad inhibitory effects against bacteria. Among AMPs, polymyxin B and E (also known as colistin) are last-resort compounds commonly used in the clinic. However, the literature presents conflicting results on the defensive role of the capsule against AMPs, and specifically, polymyxins (8–12). The exogenous addition of purified capsule significantly increases cell survival by acting as a decoy (9). On the other hand, strains with loss-of-function mutations in *wcaJ* (11, 13) or *wz*c (13) are more resistant to polymyxins.

**FIG 1:**
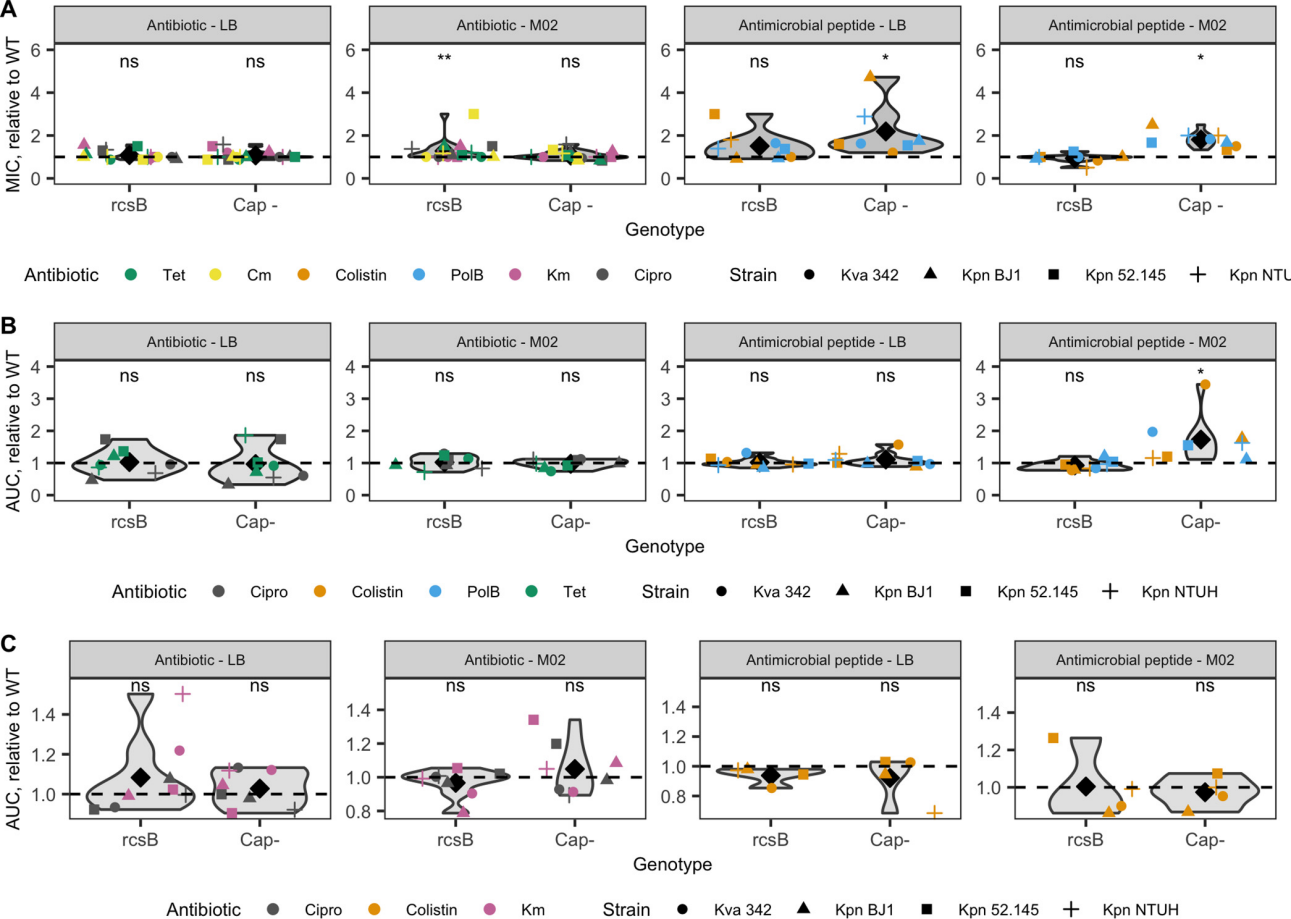
The capsule impacts the MIC and growth when the cell is challenged with antibiotics. (A) MIC relative to the wild type. Each point represents the mean across all replicates for each genotype from each strain with each antimicrobial compound. Black diamonds depict the mean. (B) Area under the curve (AUC) relative to the wild type when grown at subinhibitory concentrations (0.5× MIC) of tetracycline, colistin, polymyxin B, and ciprofloxacin. Qualitatively similar results are obtained when treated with 0.25 and 0.2 MIC (see Fig. S4 in the supplemental material). (C) AUC relative to the wild type of bacterial survival when treated with 10× MIC of kanamycin, colistin, and ciprofloxacin. Qualitatively similar results were obtained for treatment with 50× MIC. Wilcoxon rank sum two-sided test (difference from 1). *, *P* < 0.05; **, *P* < 0.01; ns, not significant (*P* ≥ 0.05). Tet, tetracycline; Cm, chloramphenicol; PolB, polymyxin B; Km, kanamycin; Cipro, ciprofloxacin. LB indicates the nutrient-rich medium, whereas M02 corresponds to the nutrient-limited medium, with glucose as the sole carbon source. Strain Klebsiella variicola 342 was not considered for the Δ*rcsB* statistical calculations, since it did not impact the capsule production (see Fig. S1 in the supplemental material). The statistics were not affected when strain K. pneumoniae CIP 52.145 and its Δ*cps* mutant were removed.

To test the impact of the capsule in antimicrobial resistance, we determined the MICs for four representative strains of the Klebsiella pneumoniae species complex (Table 1) and two types of isogenic mutants that produce a different quantity of capsule: the Δ*rcsB* mutant, which is capsulated but produces quantitatively less capsule than the wild type, and the Δ*wcaJ or Δcps* mutants, which are noncapsulated (see Fig. S1 in the supplemental material). These mutants were each compared to the respective wild type (WT). We assessed the MIC in the presence of last-resort polymyxins (polymyxin B and colistin) and four other conventional antibiotics with diverse mechanisms of action (Table S1) in a nutrient-rich (LB) and a nutrient-limited (M02) environment. Analyses of the 144 different combinations of antibiotic, strain, and environment revealed that noncapsulated strains displayed a significantly higher MIC than the WT in both media when treated with polymyxins but not the other antibiotics (Fig. 1A; Fig. S2). The difference between capsulated and noncapsulated variants was consistent across strains, but in the nutrient-limited medium, we observed that it was particularly high in the hypervirulent strain K. pneumoniae NTUH. Furthermore, in this strain, such a difference in MIC was also observed when it was exposed to kanamycin. Stepwise linear regression analyses confirmed that the MIC values were strongly affected by the type of antibiotic and the nutrient conditions, as expected, as well as by the capsule genotype (linear regression, *R*^2^ = 0.87; *P* < 0.01 for all factors; Table S3). Despite the expected differences across media in the MIC values (20, 21), the decrease in susceptibility of the noncapsulated strains was observed in both the nutrient-rich and nutrient-limited conditions, showing that it does not rely uniquely on the cellular metabolic state.

**TABLE 1:** Strains used in this study^*a*^

Strain	Species	Isolation source	ST	K serotype	O serotype	*rmpA*	Capsule mutants	Genome size (MB)	Reference
NTUH-K2044	K. pneumoniae	Liver abscess, Taiwan	ST23	K1	O1v2	*rmpA*, *rmpA*	*ΔrcsB*, *ΔwcaJ*	5.25	14
BJ1	K. pneumoniae	Liver abscess, France	ST380	K2	O1v1	*rmpA*	*ΔrcsB*, *ΔwcaJ*	5.26	15
CIP 52.145	K. pneumoniae	Indonesia	ST66	K2	O1		*ΔrcsB*, *Δcps*	5.44	16
342	Klebsiella variicola	Maize, USA	ST146	K30	O3/O3a		*ΔrcsB*, *ΔwcaJ*	5.64	17

a Kleborate (18) was used to determine the species, sequence type (ST), K serotype, O serotype, and presence of genes associated with hypermucoidy (19). All confidence values were classified as strong or very high.

The decreased susceptibility of the noncapsulated bacteria to polymyxins may have been due to a smaller effect on the growth rate or a decreased killing rate. To test this hypotheses, we first assessed the area under the curve (AUC) of the growth curves in the presence of subinhibitory concentrations of each polymyxin and of two other antibiotics, the bacteriostatic tetracycline and the bactericidal ciprofloxacin. There was no significant difference between the capsulated and noncapsulated strains in the nutrient-rich medium when they were grown with the antimicrobials (0.5× MIC; Fig. 1B). However, in the nutrient-limited medium, the noncapsulated variants grew better in the presence of polymyxins (0.5× MIC; Fig. 1B), even in the presence of larger absolute amounts of antimicrobial peptides (Table S2), compared to the capsulated strains. This growth advantage is all the more remarkable as there is a fitness cost of not producing a capsule in M02 medium (22) (Fig. S3). Qualitatively similar results were obtained when strains were grown at 0.1× and 0.25× MIC (Fig. S4). A stepwise regression model indicated that the antibiotics, strain, and then nutrients influenced the AUC, while no effect due to genotype was observed (linear regression, *R*^2^ = 0.55; Table S3).

To test whether the decreased susceptibility of noncapsulated strains was due to a decreased death rate, we measured the bacterial survival when exposed to high concentrations of antibiotics (10× or 50× MIC). As expected, the bacteria died quickly (Fig. S5). No significant differences were observed between capsulated and noncapsulated cells, irrespective of the growth medium (Fig. 1C). We conclude that the presence of the capsule does not impact the death rate at high antimicrobial concentrations. Interestingly, regrowth, and thus tolerance, was readily observed when bacterial cells were exposed to high doses of colistin in LB, as previously reported (13) (Fig. S5A). In contrast, no regrowth was observed in M02 medium. Similarly, we found surviving subpopulations of the K2 strains (K. pneumoniae BJ1 and K. pneumoniae CIP 52.145) when cells were grown in kanamycin and ciprofloxacin, in agreement with previous experiments (23, 24) (Fig. S5B).

The decreased susceptibility of noncapsulated strains supports recent research suggesting that noncapsulated variants are more resistant to polymyxins (11) and that capsule inactivation is an efficient mechanism of resistance (13). Yet several groups have independently shown that the exogenously added capsule, or other exopolysaccharides, can bind AMPs, including polymyxins (9, 25). This was interpreted as evidence that the capsule provides resistance by sequestering the AMPs, thereby potentially preventing their entrance into the cell and reducing the polymyxin killing action. In light of our experiments, we speculate that when the capsule is added exogenously, this results in titration of the AMPs, and bacteria can withstand larger doses of the antimicrobial. When the capsule is in its natural biological role, i.e., attached to the outer membrane, this results in higher concentrations of antimicrobials near the cell. The increased concentration of antimicrobial peptides near the outer membrane is particularly helpful for antimicrobial peptides like colistin, whose mode of action is to bind to lipopolysaccharides and phospholipids in the outer cell membrane, leading to the disruption of the outer cell membrane and cell lysis (26, 27). The detrimental effect of the capsule was mostly observed in low-nutrient conditions, where the capsule is most expressed (Fig. S1). We hypothesize that the slower growth in the nutrient-limited medium was associated with slower renewal of the cell wall (and capsule), which could have resulted in higher concentrations of the antimicrobial peptides than in situations of faster growth. As a result, the bactericidal action of the peptides was increased.

To conclude, our observations may have important clinical implications. First, our results confirm previously observed data (19) showing that hypervirulent strains (i.e., K. pneumoniae NTUH and BJ1), despite their hypermucoviscosity, do not seem to exhibit increased tolerance to antibiotics, as measured using the MIC. Second, the use of therapies that may target the capsule, such as conjugate vaccines (28) or phage therapy, which oftentimes results in capsule inactivation (29, 30) and potentially in reduced virulence, could be a double-edged sword. We show that it may result in decreased intrinsic susceptibility to last-resort antimicrobials, while increasing the spread of plasmid-borne antibiotic resistance (31). Finally, these results challenge the view that capsules systematically protect against antimicrobials. Instead, we advise that the effects of capsules on resistance be systematically tested, since they may depend on the type of antimicrobial agent and on the species.

### Data availability.

Data used in this study are available at the following link: https://doi.org/10.6084/m9.figshare.21400050.v1.
