# Supplementary material for: The Capsule Increases Susceptibility to Last-Resort Polymyxins, but Not to Other Antibiotics, in Klebsiella pneumoniae
Source: Antimicrob Agents Chemother. 2023 Mar 13;67(4):e00127-23. doi: 10.1128/aac.00127-23 (PMC10112221; doi:10.1128/aac.00127-23)
Supplement: Supplemental file 1 — Supplemental material. Download aac.00127-23-s0001.pdf, PDF file, 0.6 MB [file aac.00127-23-s0001.pdf]

SUPPLEMENTAL MATERIAL for.

# The capsule increases susceptibility to last-resort polymyxins, but not to other antibiotics, in *Klebsiella pneumoniae*

Francesca D'Angelo[1], Eduardo P.C. Rocha[1] and Olaya Rendueles[1]*

[1]Institut Pasteur, Université Paris Cité, CNRS UMR3525, Paris, 75015, France
*corresponding author: olaya.rendueles-garcia@pasteur.fr

## Table of Contents

SUPPLEMENTAL MATERIALS AND METHODS ....................2
SUPPLEMENTARY FIGURES ....................5
SUPPLEMENTARY TABLES ....................10

## SUPPLEMENTAL MATERIALS AND METHODS

**Bacterial strains, growth conditions and antimicrobial compounds.** Four strains from *Klebsiella* complex were used in this study (Table 1): the environmental strain *K. variicola* 342, *K. pneumoniae* BJ1, the hypervirulent *K. pneumoniae* NTUH K2044 and *K. pneumoniae* CIP 52.145 used in previous studies [8, 9]. All strains were grown at 37°C in shaking conditions, unless indicated otherwise. LB (1% tryptone, 1% NaCl and 0.5% yeast extract) and M02 (M63B1 supplemented with 0.2% glucose as sole carbon source) were used as rich and nutrient-limited medium, respectively. LB has a carrying capacity of ~ 2 x $10^9$ CFU/mL whereas M02 has one of ~ 6 x $10^8$ [31]. Chloramphenicol (Cm), tetracycline (Tet), kanamycin (Km), ciprofloxacin (Cipro), polymyxin B (PolB) and Colistin have been purchased from Sigma-Aldrich and used at the concentrations indicated in the text.

**Mutant construction.** In-frame *wcaJ* and *rcsB* deletion mutants were generated in [21], by allelic exchange. Loss-of-function mutations in *wcaJ* are among the most commonly observed in either lab-evolved [21] or in natural populations of non-capsulated *Klebsiella* [25]. This is because it is the first enzyme of the pathway, and avoids the accumulation of any unstabilizing metabolites. Despite several attempts, we did not succeed in constructing *wcaJ* mutant in CIP 52.145, nor a *manC* mutant [9]. We thus deleted the whole *cps* operon by replacing the operon by a KmFRT cassette using λ-red recombination, as described in [32]. Briefly, a KmFRT cassette was built by flanking the resistance gene by PCR two ~ 500 bp homology arms that encompass the first promoter of the capsule locus upstream *galF* and the terminator located downstream *ugd*. The cassette was electroporated into cells previously transformed with a plasmid expressing λ-red genes by virtue of pKOBEG199 plasmid, to increase recombination. To excise the KmFRT marker, the pMPIII plasmid encoding the flippase FLP that cuts at the FRT sites have been used and mutants were verified by Illumina sequencing for off-target mutations. Of note, the same results were obtained with strains carrying the deletion of one capsule gene (Δ*wcaJ*), or the deletion of the whole operon (Δ*cps*), as shown in detail in Figure S2 and Table S2. This suggests that there are no polar effects of the Δ*wcaJ* mutation on the rest of the capsule operon.

**MIC determination.** Minimal inhibition concentration (MIC) was calculated in either LB or M02 by microdilution as recommended by The European Committee of Antimicrobial Susceptibility Testing (EUCAST) guidelines

(https://www.eucast.org/fileadmin/src/media/PDFs/EUCAST_files/Breakpoint_tables/v_12.0_Breakpoint_Tables.pdf), with some modifications. Antibiotic and antimicrobial peptides stock concentrations were diluted until working concentrations. Overnight cultures were grown in either LB or M02, and adjusted to reach final concentration of 5 x $10^5$ CFU/mL. Diluted cultures and antimicrobial compounds, were mixed in a 1:1 ratio to a final volume of 100 µL in microtiter plate wells. The MIC was determined as the lowest concentration of the antibiotic that completely inhibits visible growth as judged by the naked eye after 24 hours of incubation at 37°C in static conditions. For each independent experiment, the cells were serially diluted to verify the inoculum concentration. We checked whether there was a significant correlation between inoculum concentration and MIC (Spearman's correlation, $p$-value = 0.0006, rho = 0.1935234, N = 305). However, to avoid any bias, all data presented and analyzed here are well within the range of between 5.1 and 5.9 x $10^5$ CFU/mL.

**Survival time-killing curves.** Bacterial cells were grown overnight in either LB or M02, then the morning refreshed (1/1000 in LB and 1:100 in M02) until OD ± 0.2 was reached. Antimicrobial compounds (at 10X or 50X MIC) were added to the medium (time zero). Cultures were incubated at 37°C and, at different time points, serial dilutions were prepared and plated onto LB agar plates for CFU counting. Untreated samples were plated as controls. The survival curves are expressed as $\log_{10}$ of the CFU/mL and the detention limit was 2. Data is presented as explained above.

**Growth curves.** Growth assays were performed in either LB or M02 in 96-well microtiter plates. Bacterial cells were grown as above, except for Kpn BJ1 and CIP 52.145, in M02. These two strains had large lag time in M02, with delayed exponential growth. For these, we used directly measured overnight cultures diluted 1:50 in M02. This did not impact the results. Antimicrobial compounds at sub-MIC concentrations (0.1X, 0.25X and 0.5X MIC) were mixed in a 1:1 ratio to a final volume of 200 µL. Untreated samples were grown as controls. Growth was measured as the optical density at 600 nm ($OD_{600}$) in a Tecan Spark 10M microtiter plate reader for 16 hours, every 15 minutes, and growth is presented as the Area Under the Curve (AUC) relative to the WT, as calculated by the *trapz* function in the pracma package for R.

**Capsule quantification.** The bacterial capsule was extracted using the hot phenol method as described in [33], and quantifying uronic acid contents [34]. The uronic acid concentration in

each sample was determined from a standard curve of glucuronic acid. All knockout mutants had reduced capsule production except *rcsB* mutants in *K. variicola* 342 (Figure S1).

**Statistics analyses.** All the data analyses were performed with R version 3.5.3 and Rstudio version 1.2. Statistical tests were performed with the base package stats. We used stepwise regression model to test whether antimicrobial compound, genotype, strain or environment influenced MIC or growth. All experiments were performed at least three independent times.

## SUPPLEMENTARY FIGURES

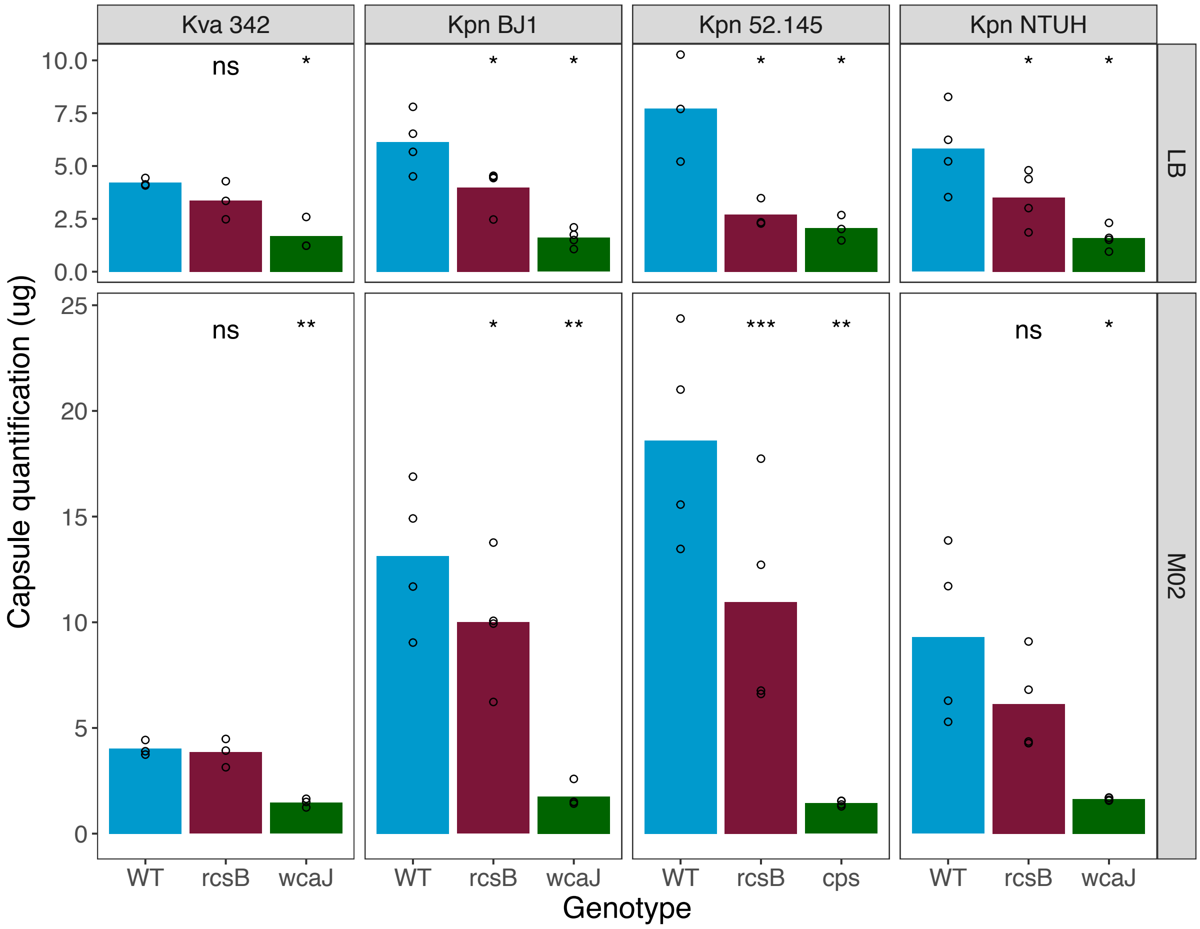


**Figure S1. Capsule production of each strain tested in this study.** WT (wild type), blue; *rcsB*, red; *wcaJ/cps*, green. Bars depict the mean and each dot constitutes an independent biological replicate. Paired two-sided t-test, difference from wild type (WT) ; * $P<0.05$, ** $P<0.01$, *** $P < 0.001$, ns not-significant.

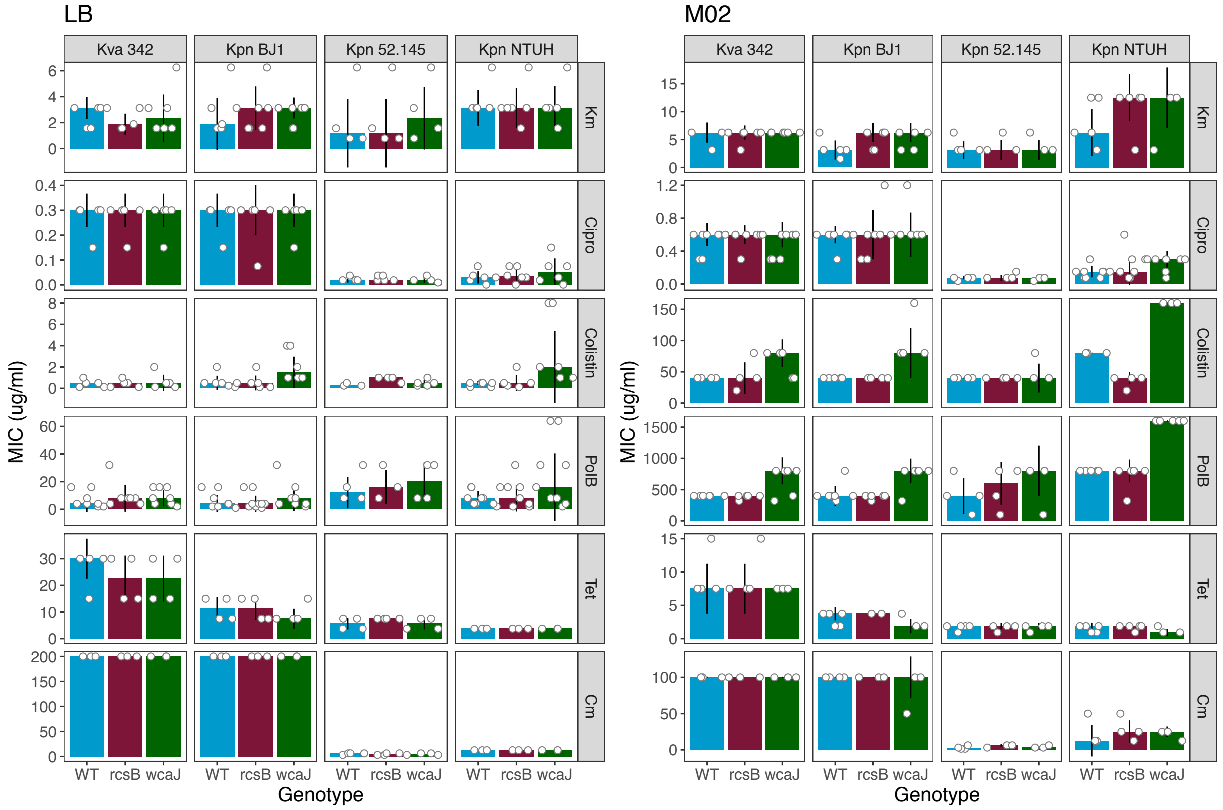


**Figure S2. MIC values for each genotype, strain and antimicrobial compound**. WT (wild type), blue; *rcsB*, red; *wcaJ/cps*, green. Bars depict the median, and error bars the standard deviation from the median. Each dot constitutes an independent biological replicate. Antibiotics: ‘Tet’ for tetracycline, ‘Cm’ for chloramphenicol, ‘PolB’ for polymyxin B, ‘Km’ for kanamycin and ‘Cipro’ for ciprofloxacin.

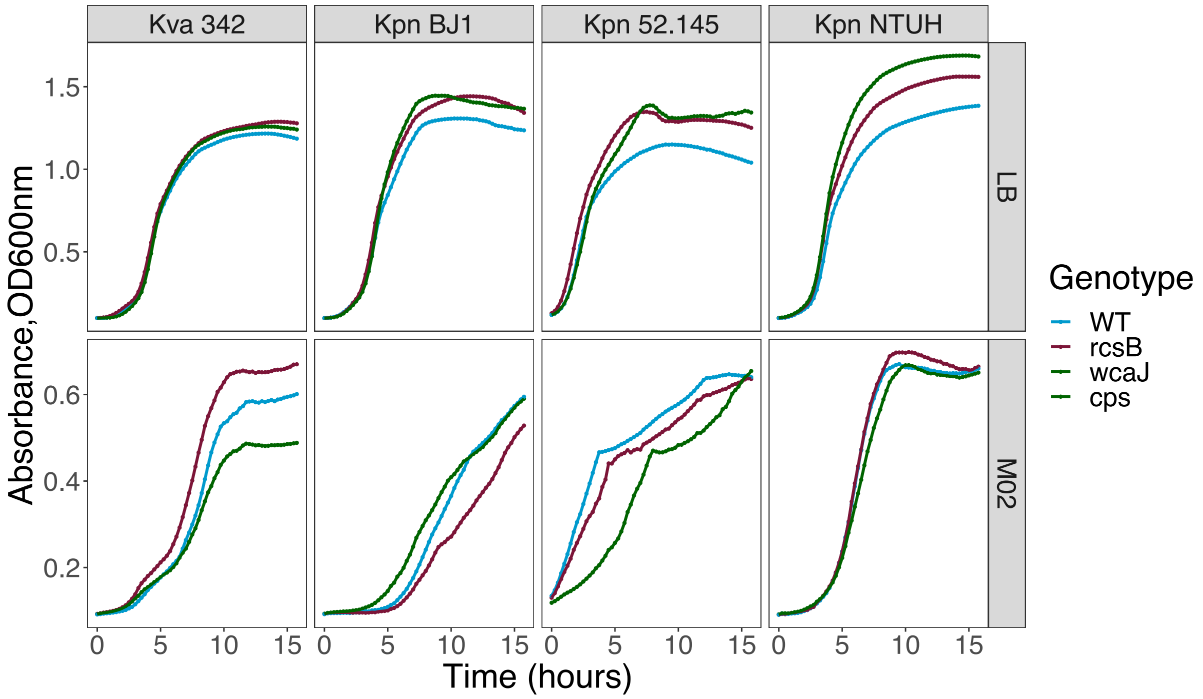


**Figure S3. Growth curves of all strains in the absence of antibiotics**. WT (wild type), blue; *rcsB*, red; *wcaJ/cps*, green. Each growth curve represents the average of at least three independent replicates. Error bars are not shown for clarity purposes.

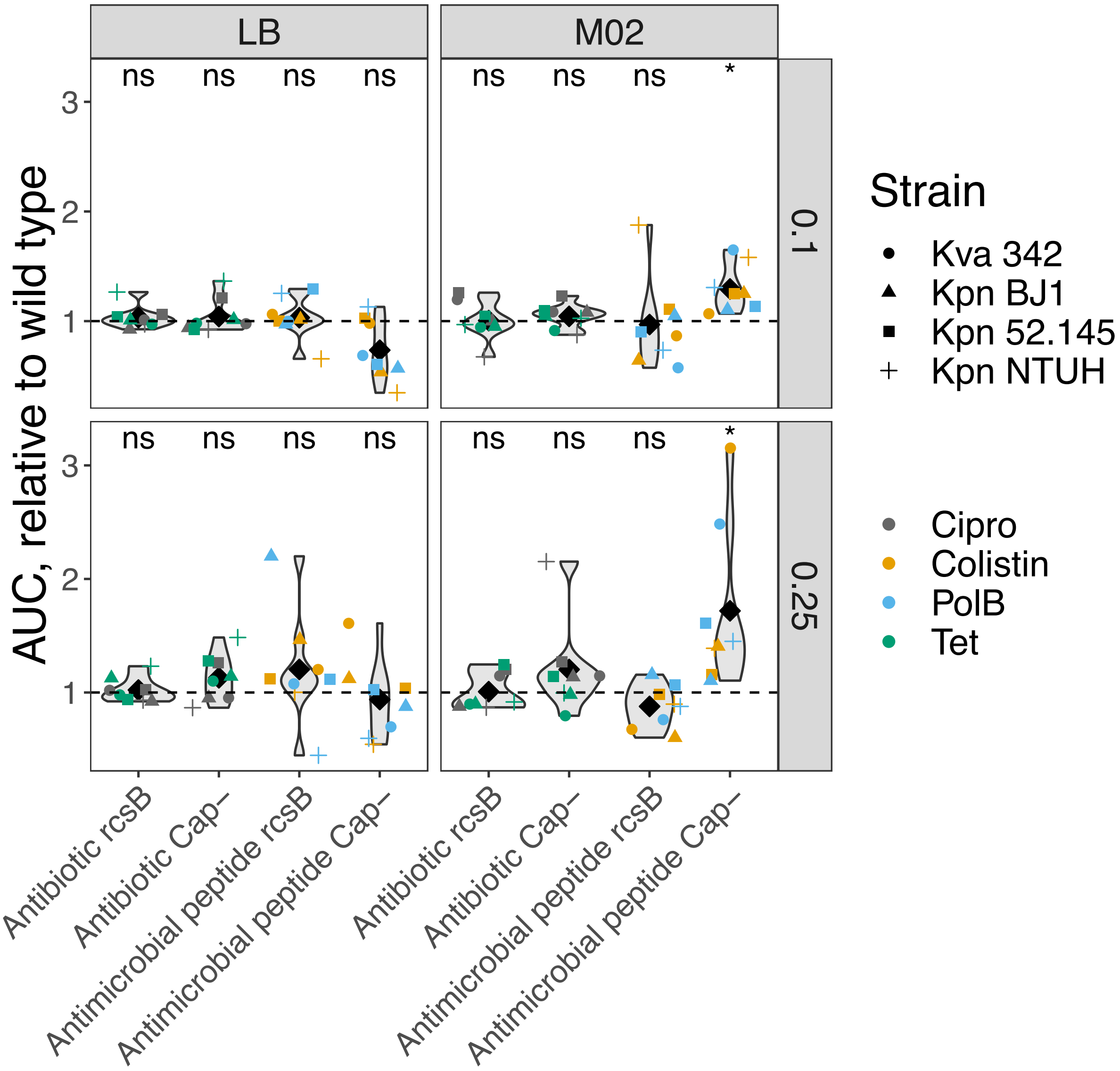


**Figure S4. Growth relative to the wild type at different MICs**. Black diamonds depict the mean. Wilcoxon sum rank two-sided test, difference from 1. * P<0.05, ** P<0.01, ns non-significant. Strain Kva 342 was not considered for *rcsB* calculations since it does not impact capsule production (Figure S1). Statistics are not affected if strain Kpn 52.145 and its *cps* mutant are removed. Antibiotics: 'Tet' for tetracycline, 'PolB' for polymyxin B and 'Cipro' for ciprofloxacin.

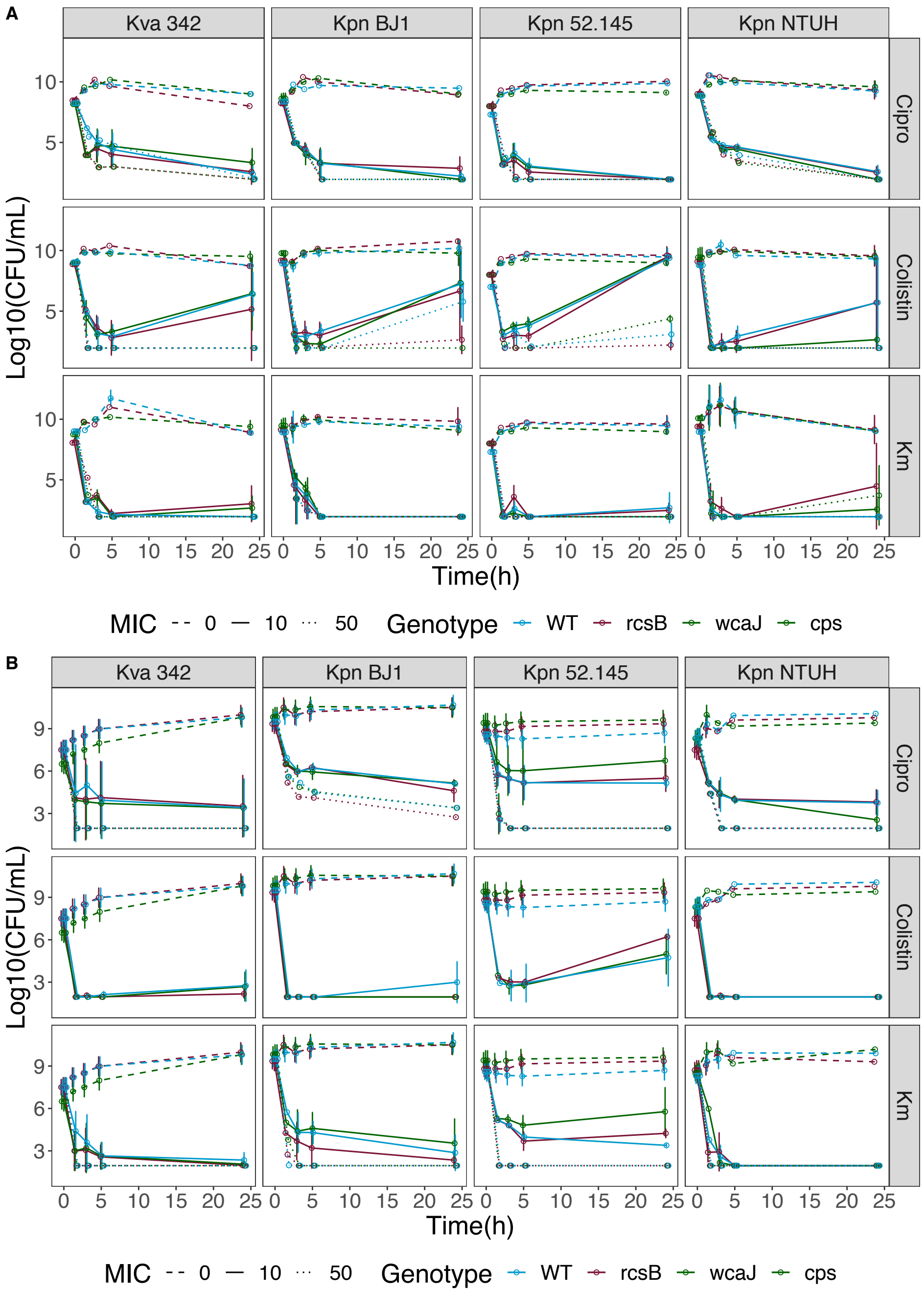


**Figure S5. The capsule does not have a role in bacterial survival.** Time-killing survival curves performed in LB (**A**) and M02 (**B**) of all the strains used in this study, exposed to 10X MIC (solid lines) and 50X MIC (pointed lines) of ciprofloxacin, colistin and kanamycin. Broken lines represent the untreated strains. Increase of bacterial cell numbers after 24 hours is indicative of the emergence of resistant clones (colistin treatment in nutrient rich media, 10X). Failure to eliminate all bacterial cells from culture could correspond to the presence of a persister sub-population, as observed strains treated with ciprofloxacin (10X) in nutrient-limited media. WT (wild type), blue; *rcsB*, red; *wcaJ/cps*, green. Three experiments have been performed independently.

## SUPPLEMENTARY TABLES

**Table S1. Antimicrobial compounds used in this study**. The clinical breaking point for each antibiotic for Enterobacterales according to EUCAST (https://www.eucast.org/clinical_breakpoints) in nutrient rich MH medium, and actualized in January 2022, is indicated. ND, not determined.

| Antibiotic type | Name | Mechanism | Action | Clinical breaking point (mg/L) |
|---|---|---|---|---|
| Antimicrobial peptides | Polymyxin B | Displaces $Ca^{2+}$ and $Mg^{2+}$ from phosphates of membrane proteins, altering membrane permeability, resulting in cell leakage | Bactericidal | ND |
| | Polymyxin E (colistin) | Disrupts LPS 3D-structure, by inserting in the fatty acid chain and expanding the outer membrane. This leads to increased permeability. | Bactericidal | R > 2 |
| Other antibiotics | Tetracycline | Diffuses through porin channels and reversibly binds to the 30S ribosomal subunit, preventing the formation of tRNA to the mRNA-ribosome complex, and blocking protein synthesis | Bacteriostatic | ND |
| | Chloramphenicol | Diffuse through the bacterial cell membrane and binds to the 50S ribosomal subunit preventing elongation of polypeptide chain | Bacteriostatic | R > 8 |
| | Kanamycin | Binds 30S causing mRNA misreading and leading to protein malformation | Bactericidal | ND |
| | Ciprofloxacin | Prevents DNA gyrase from supercoiling the bacterial DNA and prevents DNA replication | Bactericidal | R > 0.5 |

**Table S2. MICs calculated for all the strains used in this study**. Each number corresponds to the median value (reported in μg/mL) of independent experiments. (Km) kanamycin; (Cipro) ciprofloxacin; (PolB) polymyxin; (Tet) tetracycline; (Cm) chloramphenicol. Clinical break points for the determination of resistance or susceptible are as follows, calculated in MH, a nutrient-rich medium: Ciprofloxacin > 0.5 mg/L, Colistin = 2

| Medium | Species | Strain | Genotype | Km | Cipro | Colistin | PolB | Tet | Cm |
|---|---|---|---|---|---|---|---|---|---|
| LB | *K. pneumoniae* | NTUH-K2044 | WT | 3.75 | 0.037 | 0.5 | 8.4 | 3.75 | 12.5 |
| | | | *rcsB* | 3.43 | 0.047 | 0.84 | 11.3 | 3.75 | 12.5 |
| | | | *wcaJ* | 3.43 | 0.062 | 3.43 | 27.1 | 3.75 | 12.5 |
| | | BJ1 | WT | 2.6 | 0.27 | 1.12 | 7.43 | 11.3 | 200 |
| | | | *rcsB* | 3.12 | 0.26 | 1.11 | 8.86 | 11.3 | 200 |
| | | | *wcaJ* | 2.6 | 0.27 | 2.43 | 10.9 | 9.38 | 200 |
| | | CIP 52.145 | WT | 1.56 | 0.022 | 0.36 | 13.7 | 5.25 | 5 |
| | | | *rcsB* | 1.56 | 0.026 | 1.07 | 17.1 | 7.5 | 4.37 |
| | | | *cps* | 2.34 | 0.021 | 0.68 | 19 | 4.5 | 4.37 |
| | *K. variicola* | 342 | WT | 2.5 | 0.27 | 0.73 | 7.71 | 26.3 | 200 |
| | | | *rcsB* | 2.18 | 0.27 | 0.73 | 10.3 | 22.5 | 200 |
| | | | *wcaJ* | 2.81 | 0.27 | 0.9 | 10.3 | 22.5 | 200 |
| M02 | *K. pneumoniae* | NTUH-K2044 | WT | 8.12 | 0.15 | 80 | 800 | 1.5 | 25 |
| | | | *rcsB* | 10.6 | 0.25 | 40 | 700 | 1.68 | 28.1 |
| | | | *wcaJ* | 9.4 | 0.22 | 160 | 1600 | 1.31 | 21.9 |
| | | BJ1 | WT | 3.12 | 0.55 | 40 | 433 | 2.81 | 100 |
| | | | *rcsB* | 4.43 | 0.6 | 40 | 500 | 2.81 | 100 |
| | | | *wcaJ* | 4.43 | 0.7 | 96 | 833 | 2.34 | 87.5 |
| | | CIP 52.145 | WT | 3.75 | 0.075 | 40 | 340 | 1.68 | 4.06 |
| | | | *rcsB* | 3.75 | 0.09 | 40 | 500 | 1.68 | 5 |
| | | | *cps* | 3.75 | 0.068 | 48 | 660 | 1.68 | 4.37 |
| | *K. variicola* | 342 | WT | 5 | 0.5 | 40 | 371 | 7.5 | 100 |
| | | | *rcsB* | 5.6 | 0.55 | 43 | 343 | 7.5 | 100 |
| | | | *wcaJ* | 6.25 | 0.5 | 67 | 629 | 7.5 | 100 |

**Table S3. Statistic details for the generalized linear model used.** First, analyses of variance were performed to indicate significance of each factor. The formula used was as follows Y ~ Strain * Genotype * Environment * Antimicrobial compound. For the stepwise linear regression model, a linear model with all the above-mentioned predictors was established, and the stepwise regression was performed in both directions.

**MIC ($R^2$ = 0.87)**

| Step | Df | Deviance | Resid. Df | Resid. Dev | AIC | P value |
|---|---|---|---|---|---|---|
| | | | 685 | 31835995 | 7373.228 | |
| + Antimicrobial compound | -5 | 8451035 | 680 | 23384960 | 7171.594 | <0.0001 |
| + Environment | -1 | 3394237.7 | 679 | 19990722 | 7066.012 | <0.0001 |
| + Genotype | -2 | 258433.2 | 677 | 19732289 | 7061.086 | 0.0005 |

**Growth rate at subinhibitory concentrations (MIC = 0.5X) ($R^2$ = 0.55)**

| Step | Df | Deviance | Resid. Df | Resid. Dev | AIC | P value |
|---|---|---|---|---|---|---|
| | | | 182 | 14.294773 | -464.5754 | |
| + Antimicrobial compound | -3 | 4.00705 | 179 | 10.287723 | -518.7719 | <0.0001 |
| + Strain | -2 | 2.032117 | 177 | 8.255606 | -555.0426 | <0.0001 |
